# Supplementary material for: Does managerial short-termism always matter in a firm's corporate social responsibility performance? Evidence from China
Source: Heliyon. 2023 Mar 13;9(3):e14240. doi: 10.1016/j.heliyon.2023.e14240 (PMC10025896; doi:10.1016/j.heliyon.2023.e14240)
Supplement: Multimedia component 1 [file mmc1.pdf]

COPYLEAKS

23manuscript-2.docx

Scanned on: 7:59 February 23, 2023 UTC

6.8%	35	7379
Overall similarity score	Results found	Total words in text

	Word count
Identical	104
Minor Changes	106
Paraphrased	289
Omitted	0

23manuscript-2.docx

Scanned on: 7:59 February 23, 2023 UTC

Results

The results include any sources we have found in your submitted document that includes the following: identical text, minor changed text, paraphrased text.

Construction and Analysis of Corporate Social Responsibilit...
https://www.hindawi.com/journals/scn/2022/3306735/
1%

35178-corporate-governance-and-firm-value-the-impact-of-c...
https://jps.scholasticahq.com/api/v1/articles/35178-corporate-governance...
35178-corporate-governance-and-firm-value-the-impact-of-chinese-c...
1%

Does corporate governance matter in competitive industries?
http://www.columbia.edu/~xg2285/Pmc.pdf
Pmc.pdf
1%

EFMA2018_0050_fullpaper.pdf
https://www.efmaefm.org/0EFMAMEETINGS/EFMA%20ANNUAL%20MEETIN...
EFMA2018_0050_fullpaper.pdf
0%

The Economic Effects of COVID-19 and Credit Constraints: Ev...
https://docs.iza.org/dp13629.pdf
dp13629.pdf
0%

New productivity drivers: Revisiting the role of digital capit...
https://www.econstor.eu/bitstream/10419/223083/1/wiiw-wp-178.pdf
wiiw-wp-178.pdf
0%

Sustainability Performance and the Cost of Capital
https://mdpi-res.com/d_attachment/ijfs/ijfs-10-00063/article_deploy/ijfs-1...
ijfs-10-00063-v2.pdf
0%

Park_89.pdf
http://www.law.harvard.edu/programs/olin_center/fellows_papers/pdf/Par...
Park_89.pdf
0%

IDENTICAL

Text that is exactly the same.

MINOR CHANGES

Text that is nearly identical, yet a different form of the word is used. (i.e 'slow' becomes 'slowly')

PARAPHRASED

Text that has similar meaning, yet different words are used to convey the same message.

Unsure about your report?

The results have been found after comparing your submitted text to online sources, open databases and the Copyleaks internal database. If you have any questions or concerns, please feel free to contact us atsupport@copyleaks.com

Click here to learn more about different types of plagiarism

23manuscript-2.docx

Scanned on: 7:59 February 23, 2023 UTC

**Quantify_the_QE_Todorov.pdf**<br>*https://ktodorov.com/files/research/Quantify_the_QE_Todorov.pdf*<br>Quantify_the_QE_Todorov.pdf	0%
**The relationship between immigration to Australia and the ...**<br>*https://www.pc.gov.au/inquiries/completed/migrant-intake/report/migrant...*<br>migrant-intake-supplementa.docx	0%
**Help in a Foreign Land: Internationalized Banks and Firms' E...**<br>*https://docs.iza.org/dp15458.pdf*<br>dp15458.pdf	0%
**Sustainability | Free Full-Text | Trends in Corporate Social R...**<br>*https://www.mdpi.com/2071-1050/13/15/8640*	0%
**legeff_16march2018_final.dvi**<br>*https://www.nber.org/system/files/working_papers/w24442/w24442.pdf*<br>w24442.pdf	0%
**Foreign investors and stock price crash risk: Evidence from ...**<br>*https://www.ncbi.nlm.nih.gov/pmc/articles/PMC7524542/*	0%
**wku_etd001_cbpm01_000668.pdf**<br>*https://wkuwire.org/bitstream/20.500.12540/766/1/wku_etd001_cbpm01_...*<br>wku_etd001_cbpm01_000668.pdf	0%
**Corporate Social Responsibility, Internal Controls, and Stock...**<br>*https://pdfs.semanticscholar.org/7100/74f2d4fd63be0dc9d35eef74b1390...*<br>74f2d4fd63be0dc9d35eef74b139048e4a44.pdf	0%

23manuscript-2.docx

Scanned on: 7:59 February 23, 2023 UTC

**How Much Are People Willing to Pay for Clean Air? Analyzin...**<br>*https://www.ncbi.nlm.nih.gov/pmc/articles/PMC8508453/*	0%
**Microsoft Word - Media_Strikes_14march2011_PaulWooleey....**<br>*https://www.parisschoolofeconomics.eu/IMG/pdf/jperess_1105.pdf*<br>jperess_1105.pdf	0%
**EVJ_PDF_Banner**<br>*https://cpb-us-w2.wpmucdn.com/u.osu.edu/dist/0/30211/files/2016/04/C...*<br>Corp-acquisitions-diversification-and-the-firms-life-cycle_JF2016-xqbw...	0%
**Can the low-carbon city pilot policy promote firms' low-carb...**<br>*https://journals.plos.org/plosone/article/file?id=10.1371/journal.pone.027...*	0%
**h1b.pdf**<br>*https://gspp.berkeley.edu/assets/uploads/research/pdf/h1b.pdf*<br>h1b.pdf	0%
**pmc18..dvi**<br>*https://www.ecgi.global/sites/default/files/working_papers/documents/SSR...*<br>SSRN-id1006118.pdf	0%
**gibsonbrandonglossnerkruegermatossteffenfinal.pdf**<br>*https://www.ecgi.global/sites/default/files/working_papers/documents/gib...*<br>gibsonbrandonglossnerkruegermatossteffenfinal.pdf	0%
**Managerial Short-Termism and Investment: Evidence from ...**<br>*https://academic.oup.com/rof/article/24/2/305/5529965*	0%

23manuscript-2.docx

Scanned on: 7:59 February 23, 2023 UTC

**Essays on Sustainable Finance and Corporate Social Respon...**<br>*https://jlupub.ub.uni-giessen.de/bitstream/handle/jlupub/713/BofingerYa...*<br>BofingerYannik-2022-03-17.pdf	0%
**Learning-Curve_AAA.pdf**<br>*https://s3.eu-central-1.amazonaws.com/ng-submission-additional-files/AA...*<br>Learning-Curve_AAA.pdf	0%
**Paper_1-1.pdf**<br>*https://cba.lmu.edu/media/lmucollegeofbusinessadministration/departm...*<br>Paper_1-1.pdf	0%
**Sustainability | Free Full-Text | Corporate Social Responsibil...**<br>*https://www.mdpi.com/2071-1050/10/5/1675*	0%
**Essays on Corporate Social Responsibility and Sustainable Fi...**<br>*https://jlupub.ub.uni-giessen.de/bitstream/handle/jlupub/617/RockBjoern...*<br>RockBjoern-2022-01-27.pdf	0%
**New Housing Registrations as a Leading Indicator of the BC ...**<br>*https://www.bankofcanada.ca/wp-content/uploads/2016/02/sdp2016-3.pdf*<br>sdp2016-3.pdf	0%
**Death and contagious infectious diseases: Impact of the CO...**<br>*https://www.ncbi.nlm.nih.gov/pmc/articles/PMC7144859/*	0%
**abnormal-air-pollution-crime.pdf**<br>*https://drderemer.github.io/papers/abnormal-air-pollution-crime.pdf*<br>abnormal-air-pollution-crime.pdf	0%

COPYLEAKS

23manuscript-2.docx

Scanned on: 7:59 February 23, 2023 UTC

**18-19-03.pdf**<br>*https://www.economics.uci.edu/research/wp/1819/18-19-03.pdf*<br>18-19-03.pdf	0%
**DJRW_CustomerCapital_AssetPricing_Paper.pdf**<br>*http://finance-faculty.wharton.upenn.edu/wdou/wp-content/uploads/sites...*<br>DJRW_CustomerCapital_AssetPricing_Paper.pdf	0%
**Sustainability | Free Full-Text | Do CSR Ratings Converge in ...**<br>*https://www.mdpi.com/2071-1050/11/14/3921*	0%

COPYLEAKS

COPYLEAKS

23manuscript-2.docx

Scanned on: 7:59 February 23, 2023 UTC

Scanned Text

Your text is highlighted according to the plagiarism types that where found, as shown above.

IDENTICAL MINOR CHANGES PARAPHRASED

1. Introduction
2. Literature review and hypothesis development2.1 Relevant studies on managerial short-termism
2.2 Relevant studies on CSR
2.3 The association between managerial short-termism and CSR performance
2.4 Moderating effect2.4.1 Moderating role of internal corporate governance
2.4.2 Moderating role of product market competition
2.4.3 Moderating role of analyst coverage
2.4.4 Moderating role of ownership structure

3. Data, variable measurement and methodology3.1 Data
3.2 Variable measurement3.2.1 CSR performance
3.2.2 Managerial short-termism
3.2.3Moderation variables(1) Internal corporate governance
(2) Product market competition
(3) Analyst coverage
(4) Ownership structure

3.2.4 Other control variables
3.2.6 Methodology

4. Results4.1 Baseline results
4.2 Endogeneity issue
4.3 Random forest regression

5. Possible channels
6. Heterogeneity Checks
7. Further analysis
8. Conclusions, policy implications and limitations8.1 Conclusions
8.2 Policy implications
8.3 Limitations

Appendix A
Appendix B

Does managerial short-termism always matter in a firm's corporate social responsibility performance? Evidence from China

Xiaohui Xu Jun Yang*
(School of Economics and Management, Zhejiang Sci-Tech University
Hangzhou, China)

Abstract
Using data on Chinese A-share listed firms from 2008 to 2017, we explore how corporate social responsibility (CSR) performance is affected by managerial short-termism and what factors influence the association between the two. First, by employing text analysis in conjunction with machine learning, we construct a new managerial short-termism indicator. Using panel fixed models, we find that managerial short-termism has an adverse impact on CSR performance, and the results are consistent in a series of robustness checks. The heterogeneous test results show that the negative effect is significant only for firms with lower internal corporate governance, for firms in less competitive industries, for firms with less analyst attention, and for state-owned enterprises (SOEs). Additionally, a better institutional environment weakens the negative impact of managerial short-termism on CSR performance. The findings shed light on policy implications for emerging countries.

Keywords: Managerial short-termism, CSR performance, Random forest regression
JEL classifications:

1. Introduction

Companies in China are increasingly paying attention to social responsibility to maintain sustainable development. The Rankins CSR Ratings (RKS) system discloses that an increasing number of Chinese A-share listed companies disclose CSR performance. This number increased from 314 in 2008 to 851 in 2017, and the average CSR performance increased from 29.80 in 2008 to 42.51 in 2017. These results indicate a trend toward an increasing number of companies promoting social responsibility as a competitive advantage [1]. Nevertheless, the gap between companies' levels of CSR performance is significant; for example, in 2017, the minimum and maximum levels of CSR performance were 18.44 and 89.00, respectively.1

Extensive studies have investigated sources of CSR performance heterogeneity. One stream of research favors a focus on the heterogeneous nature of management characteristics, especially managerial demographic characteristics, such as age, education, and work experience [2-5]. In accordance with upper echelon theory, the personal traits of top executives affect both their behavior and their strategic decisions [6,7], thereby affecting the goals, behavior, and results of the enterprise [8]. In line with this stream, we intend to introduce another innate personal trait, time orientation, into the framework of CSR performance determinants. According to Hofstede's cultural dimensions theory, time orientation is classified into two different classifications: long-term orientation and short-term orientation. Short-term orientation, termed short-termism, is defined as the excessive focus of corporate executives on short-term quarterly earnings [9-11]. Considering that agency theory, managerial opportunism theory, and socially responsible investments were historically focused on long-term considerations [12], we aim to address the impact of managerial short-termism on CSR performance, which few studies have explored.

Hence, firstly, we develop a new indicator of managerial short-termism. Currently, scholars typically use questionnaire rating methods [13,14] or financial indicators such as the proportion of short-term investments [15,16] to estimate managerial short-termism levels. Existing measures have several drawbacks, such as low response rates and subjective cognitive biases in responses [17], while financial indicators only capture behaviors that are observed after the event, not actual perceptions of management [18]. We developed the managerial short-termism index using the dictionary method introduced by Brochet et al in 2015 [19, 20]. Researchers agree that human characteristics can be understood by examining the types and frequency of words used in the language of the subjects [21,22]. We employ text analysis in conjunction with machine learning to identify short-term horizon words used in management discussion and analysis (MD&A) in annual reports for Chinese A-share listed companies, like those identified by Li in 2010 [23]. We then use the dictionary method to construct indicators of managerial short-termism.

After developing managerial short-termism index, we turn to answer the following questions: (1) Whether managerial short-termism influences the corporate social responsibility performance of enterprises? (2) If yes, how does managerial short-termism affect an organization's CSR performance? (3) What factors influence the relation between managerial short-termism and corporate social responsibility? Accordingly, this paper has the following contributions. First, we develop a new methodology to measure managerial short-termism more objectively and directly. Second, we extend the research on the CSR performance drivers. We find that managerial short-termism plays an adverse

role in CSR performance. Third, we enrich the research on managerial short-termism. We introduce the managerial short-termism into the framework of CSR performance. Fourth, our results shed light on the important role of internal corporate governance, product market competition, analyst coverage, as well as ownership structure and hence may have important policy implications for policymakers in developing countries. Our heterogeneous tests show that the negative effect of managerial short-termism is significant only for firms with weak internal corporate governance, for firms in less competitive industries, for firms with less analyst coverage and for non-SOEs. In addition, an increasing institutional quality is beneficial to reducing the adverse impact of managers' short-termism.

1 The original data are from the RKS system.

The rest of the paper is developed as follows. Section 2 summarizes the literature and develops hypotheses. Data and variables are described in Section 3. The basic empirical results are presented in Section 4. Section 5 examines further channels that support the conclusion. A robustness test is conducted in Section 6, and a conclusion is provided in Section 7.

2. Literature review and hypothesis development

2.1 Relevant studies on managerial short-termism

As social psychologists have noted, people have different perspectives about the past, present, and future [24]. It is widely recognized that time orientation is an innate, inborn, and subconscious process, but it can also be affected by environmental factors [25, 26]. People's time orientation affects their choice and attainment of social goals as well as their emotional, cognitive, and behavioral motivations [27, 28]. In the field of management, the concept of time orientation refers to a manager's preference for viewing the past, present, or future when making strategic decisions [29, 30]. The time orientations of managers can be classified as long-term and short-term [31]. Short-termism refers to actions that increase current returns [32-35]. Surveys have found that most top managers are prone to achieve short-term objectives [36].

According to agency theory as well as managerial opportunism theory, managers concerned with their reputations on the job prefer short-term investments that boost short-term performance measures quickly to enhance their reputation [34,37]. In a similar vein, it is demonstrated that even in a fully efficient market, managers may behave myopically if they care about stock prices over a near-term horizon [9, 38]. Holmstrom and Costa in 1986 proposed a similar model where superiors learn about talent by observing project performance [39,40]. A series of studies investigated that managerial short-termism leads to short-sighted value reducing actions [32, 41, 42].

2.2 Relevant studies on CSR

Researchers have investigated the definition of corporate social responsibility [43, 44], measurement of CSR [46], the correlation between CSR and financial performance [45], and the benefits of CSR participations [47,48]. CSR is the responsibility of businesses toward their stakeholders, the environment, and society [49,50].

The literature has identified two motivations for CSR: self-interest [51] and long-term firm value [52]. First, according to agency theory [53], CSR engagement is an agency problem [45]. Managers tend to overinvest in CSR to enhance their own private benefits [54]. Second, stakeholder theory asserts that a firm can be viewed as a set of interdependent relationships among stakeholders [55], including investors, customers, employees, etc [56]. A firm's success depends on its ability to comply with stakeholders' expectations and to meet their needs. CSR can provide firm benefits via an increase in customer awareness [57, 58], stakeholder engagement [59, 60], long-run benefit sharing [61, 62]. Empirical studies have further suggested that CSR can generate competitive advantages [63, 64], increase corporate reputation [65, 66], and upgrade firm value [67, 68]. Hence, based on stakeholder theory, the motivation for CSR is long-term firm value.

Previous studies have also focused on the determinants of CSR performance, such as financial slack [69], the cost of equity capital [70], corporate governance structures [54, 71], shareholder activism [72], local community pressure [73], customer monitoring [74], and media pressure [75]]. Recent scholarly work has started to focus on the role of top managers [76]. CEOs also can affect firms' CSR policies and determine whether and how to conduct CSR activities in response to a host of stakeholder interests [77, 78]. For example, these studies have examined such influences as CEO

compensation structure [79], CEO career horizon [80] and tenure [81], CEO hubris [82] and narcissism [83], CEO moral identity [84], and CEO political ideology [85, 86] to explain the magnitude of CSR engagement by firms. However, few studies focus on the relation between managerial short-termism and CSR performance.

2.3 The association between managerial short-termism and CSR performance

Considering different motivations for CSR, we argue that there might be two opposite relationships between managerial short-termism and CSR performance [87,88]. First, if the motivation for CSR is self-interest, managers will overinvest in CSR to obtain the private benefits [45]. Accordingly, managers can overinvest in CSR activities to enhance their reputation or ethicality and extend their discretionary powers over corporate resources [45, 53].

Second, stakeholder value creation theory suggests that managers should treat CSR activities as long-term investments if CSR is motivated by the creation of long-term value for the company. Supporting the interests of other stakeholders, such as providing generous benefits to employees, is often in the best interests of shareholders [89, 90]. In a similar manner, CSR can enhance a company's reputation [91, 92]. The engagement of stakeholders through CSR could potentially reduce the likelihood of managers engaging in short-term opportunistic behavior [93, 94]. Thus, managers will reduce overinvestment in CSR activities if the motivation for CSR is long-term firm value [95].

Based on the above discussion, we propose Hypothesis 1 as follows:

Hypothesis 1a: If the motivation for CSR is self-interest, managerial short-termism plays a positive role in CSR performance.

Hypothesis 1b: If the motivation for CSR is long-term firm value, managerial short-termism draws a negative effect on CSR performance.

2.4 Moderating effect

2.4.1 Moderating role of internal corporate governance

Agency theory documented how to maximize the value of shareholders through monitoring agents [96]. Due to information asymmetry, managers are self-interested unless they are properly monitored[97]. Against this backdrop, effective corporate governance that is designed to solve this agency problem, i.e., align executives with shareholder interests, should discourage CSR-related activities unless this investment would benefit financial performance. Hence, if the motivation for managers' CSR participation is self-interest, then effective corporate governance would discourage CSR activities.

According to stakeholder theory [98], effective corporate governance contributes to improving CSR performance. It is confirmed that firms with better corporate governance quality tend to invest more in CSR activities in the USA [99], this notion is also supported based on a sample from Europe [100]. Moreover, companies with better governance are often found to be more socially responsible [101]. Hence, if the motivation for CSR is long-term firm value, then the role of corporate governance is positive.

Thus, we propose hypothesis 2a and 2b as following:

Hypothesis 2a: If the motivation for CSR is self-interest, higher internal corporate governance weakens the positive effect of managerial short-termism on CSR performance.

Hypothesis 2b: If the motivation for CSR is long-term firm value, higher internal corporate governance weakens the negative effect of managerial short-termism on CSR performance.

2.4.2 Moderating role of product market competition

Managers take the condition of product market competition into account in all their strategic decisions, including decisions about corporate social responsibility [102]. There are two views proposed in the literature regarding the role of product market competition on a firm's CSR engagement.

First, from the strategic view of CSR, firms engage in CSR activity are to benefit economically. Thus, firms in competitive industries are more like to engage in socially responsible activities to gain a competitive edge[103]. Firms in competitive industries are more socially responsible [104]. Hence, if the motivation for managers' CSR participation is long-term firm value, then higher product market competition would encourage CSR activities.

Second, if the motivation for CSR is self-interest, firms in competitive industries have incentives to engage less in socially responsible activities. In a less competitive environment, the information asymmetry problem is severe; therefore, managers can easily pursue private interests. It is documented that external supervision helps to reduce the agency problem of managerial short-termism [105]. Managerial overinvestment in CSR activities may not be detected in less-competitive industries because of the low comparability of their performance to that of other firms because there are no or few

competing firms in the same industry. Thus, managers could enhance their reputation or ethicality and extend their discretionary power over corporate resources by investing in CSR activities in less-competitive industries. Therefore, based on agency theory, if the motivation for CSR is self-interest, then higher product market competition would discourage CSR activities.

Therefore, we obtain the following hypothesis:

Hypothesis 3a: If the motivation for CSR is self-interest, higher product market competition weakens the positive association between managerial short-termism and CSR performance.

Hypothesis 3b: If the motivation for CSR is long-term firm value, higher product market competition weakens the negative correlation between managerial short-termism and CSR performance.

2.4.3 Moderating role of analyst coverage

The role of analyst coverage in the relationship between managerial short-termism and CSR relates to the motives of CSR engagements [106]. Agency theory proposes that analyst coverage can improve information transparency and decrease managers' self-interested behaviors [107]. The information transparency is related to firms' CSR decisions [108,109]. The growth of analyst coverage heightens media coverage, which in turn increase firms' CSR information exposure to stakeholders [53]. Analysts' coverage can alleviate agency problems and enhance internal corporate systems [109]. Managers' information is more likely to be exposed to investors, and enterprises are more likely to be supervised by stakeholders [110], which also limits managers' short-termism behavior [111].

Therefore, we obtain the following hypothesis:

Hypothesis 4a: If the motivation for CSR is self-interest, higher analyst coverage weakens the positive effect of managerial short-termism on CSR performance.

Hypothesis 4b: If the motivation for CSR is long-term firm value, higher analyst coverage weakens the adverse effect of managerial short-termism on CSR performance.

2.4.4 Moderating role of ownership structure

Existing research has found that ownership plays a vital role in organizational decision making [112] such as research and development (R&D) investment [113], and innovation [114] decisions. Considering corporate social actions are a form of investment [50], ownership should be involved in

the firm's CSR performance.

First, in China, compared to state-owned enterprises (SOEs), the owners and managers of non-state-owned enterprises (non-SOEs) are always the same one. When insiders become shareholders, the insiders' and shareholders' interests converge through "incentive alignment" mechanisms [53,115]. A high level of insider ownership leads to strategic decisions that are consistent with shareholders' long-term interests [116,117]. Therefore, managers in non-SOEs are likely to be supportive of CSR. A second consideration is that, information transparency can affect investor behavior [118]. For state-owned companies, there is a severe problem of information asymmetry; consequently, managers will be able to pursue their private interests without being detected [119]. Moreover, managers in SOEs have a greater concern for their career prospects. Self-interest often drives managers to overinvest in CSR activities.

Therefore, we obtain four hypotheses as follows:

Hypothesis 5a: If the motivation for CSR is long-term firm value, the adverse impact of managerial short-termism on CSR performance is weaker for non-SOEs, compared to SOEs.

Hypothesis 5b: If the motivation for CSR is self-interest, the positive impact of managerial short-termism on CSR performance is weaker for non-SOEs, compared to SOEs.

3. Data, variable measurement and methodology

This section outlines the corporate social responsibility performance data used in the empirical analysis as well as how the managerial short-termism indicator was constructed. In addition, we introduce the estimation of variables, including the internal corporate governance index, product market competition, analyst coverage, and ownership structure. The rest of the control variables, including finance feature variables and management characteristic variables, are described.

3.1 Data

Considering the availability of data on firm-level CSR, we choose the Chinese A-share listed firms as the initial research sample, and the sample period is from 2008 to 2017. The following three datasets are adopted. First, the China Stock Market and Accounting Research (CSMAR) database records listed firms' financial and management data. Second, the Rankins Corporate Social Responsibility Ratings (RKS) database reports detailed information on corporate social responsibility performance. Third, the cninfo.com website has documents for listed companies' annual financial reports2. We use the stock code to merge the three data sources in a given year and then exclude samples that satisfy one of the following criteria: financial and insurance industry3; ST, ST*, PT and

company observations less than two years [120], as well as firms with missing or negative sales, assets, or CSR performance. Finally, we obtain 4,913 firm-year observations.

3.2 Variable measurement

3.2.1 CSR performance

We get CSR data from Rankins Corporate Social Responsibility Ratings (RKS) [94,121,122]. The original CSR score ranges from 1 to 100. The higher the score, the better the CSR performance. In this paper, the score is the natural logarithm of the total score of the CSR performance[121].

3.2.2 Managerial short-termism

We develop a managerial short-termism index based on the following steps [20]. First, we download the annual financial reports of all A-share listed companies from 2007 to 2018 from the

2 http://www.cninfo.com.cn/new/index.

3 Industry classification codes are determined by a company's letter-plus-first-digit code from the China Securities Regulatory
Commission in 2001.

cninfo website4. Second, we transform PDF documents into TXT documents using the WinGo financial text data platform5, eliminate all tables through text recognition, and then delete scanned and missing documents6. Third, we extract the management discussion and analysis (MD&A) section from the annual financial report and use the WinGo financial text segmentation system to divide it and turn the unstructured text data into word vectors based on the Chinese general dictionary Jieba and the English Chinese Dictionary of modern finance and accounting. Fourth, we calculate the word frequency of the word set corresponding to the managerial short-termism index. Specifically:

(1) We read 500 MD&A files in search of key phrases that refer to a firm's strategy and investment decisions [20, 23], and then identify ten words referring to the short term as the seed word set of "short-termism" in MD&A, including "day (s or daily)", "month (-s or -ly)", "within a year", "as soon as possible", "immediately", "immediately", "trend", "chance", "pressure", and "challenge"7. The 10 keywords satisfy the criterion that the average score is under 3 [20].

(2) we use the CBOW model (continuous bag of words model) in Word2vec to train the Chinese annual financial report corpus [123,124]. The specification is (1) as following [125]:

(1)

Where C represents the corpus, stands for the head word, and represents the context of the headword. By maximizing the above objective function, we obtain the Word2vec word vector corresponding to the central word. Then, the similar words of the central word can be obtained by calculating the vector similarity.

(3) By inviting three industry and academic experts and comparing the MD&A text samples, we determine that the word set contains 43 "short-termism" words (see Appendix A). Then, based on the dictionary method, we calculate the proportion of the total word frequency of "short-termism" vocabulary in the total word frequency of MD&A and multiply it by 100 to obtain the managerial short-termism indicator. The larger the value of the managerial short-termism indicator is, the shorter the termism of managers.

3.2.3Moderation variables

(1) Internal corporate governance

We employ the internal governance variable from Yu et al's corporate governance index CGI [126]. This index describes the internal governance based on publicly information that contains 43 governance attributes.

(2) Product market competition

To measure production market competition, we use the Herfindahl-Hirschman index (HHI), combining the sales-based square market shares of all firms in an industry. The equation is (2):

4 http://www.cninfo.com.cn/new/index.

5 http://www.wingodata.com.

6 Data processing is as follows. In table processing, the table in the annual report becomes a text box after conversion, so its
content cannot be queried or analyzed directly. Moreover, because most of the content in the table is numbers, some text content
is seriously templated and contains less incremental text information.

7 In Chinese, these key words mean "天内" , "数月" , "数周" ,"年内" , "尽快" , "立刻" , "马上" ,

"之际" ,"契机" ,"压力" and "考验" .

(2)
Where is the market share of firm i in industry j in year t, and denotes the number of firms in industry j in year t. We exclude firms whose sales are either missing (negative) or above (above the top one percentile) or below (below the bottom one percentile) the median levels to limit the influence of outliers. In addition, we exclude industries with fewer than five companies. Sector classification codes are determined by a company's letter-plus-first-digit code from the China Securities Regulatory Commission (CSRC) in 2001. 8

(3) Analyst coverage

The data of analyst coverage is from CSMAR. We average the earnings forecasts for each fiscal year and firm [106,107]. Analyst coverage is the natural logarithm of one plus this measure [127].

(4) Ownership structure

The ownership structure data is from the CSMAR dataset [128]. It is state-owned if it is recorded in the CSMAR data basement as state-owned, otherwise non-SOEs. In our paper, ownership structure index is a dummy variable. When a company is SOE, the value is "1"; otherwise, "0" [119].

3.2.4 Other control variables

Similar to previous studies, we control variables as follows:

First, due to this higher attention and to lower relative activity costs, larger organizations are expected to undertake more CSR activities [50,129,130]so we include firm size as a control variable. Firm size is measured by the natural logarithm of a firm's total assets.

Second, if long-term perspective incentives dominate, then CEOs with larger inside debt holdings are expected to engage in more CSR [93]. Alternatively, CEOs with large inside debt holdings tend to commit to more CSR investment [131]. Finally, inside debt can discourage firm managers from diverting firm resources to overinvest in CSR. In this paper, debt is measured by dividing total liabilities by shareholder equity [132,133].

Third, firm growth is one of the considerations in investment decisions. High-growth companies have many opportunities for engaging in CSR activities [134,135]. Firm growth is measured by the ratio of profit to total assets.

Fourth, the literature documents the presence of women in power positions allows them to be more objective toward the existing CSR activities, and they tend to perform more scrutiny of general CSR activities [136]. Thus, the improvement in gender diversity may improve CSR performance [137]. Hence, we control gender diversity in the chairperson; female chief executive officers are coded as "1" and "0" otherwise [138].

Fifth, according to existing findings [139], the presence of independent directors not only reduces agency conflicts but also improves the effective monitoring of corporate decision-making processes [140,141]. In our paper, independent director index is a dummy variable. When the CEO and the chairperson are the same, the value is "1"; otherwise, "0". We winsorize all continuous variables at the upper and lower 1% levels.

3.2.5 Statistical analysis

Descriptions of the main variables are given in Table 1, including growth, size, debt, and HHI. We

8 The number of companies in some industries would be too low to use finer industry classification codes.

also examine the correlation between managerial short-termism and CSR performance in unreported tests. The correlation between short-termism and CSR performance is -0.081 for the whole sample, which is significantly different from zero. It indicates that the relation between managerial short-termism and CSR performance is negative.

[Insert Table 1 here]

3.2.6 Methodology
To test the impact of managerial short-termism on CSR performance (H1), we develop following specification:
(3)
Where CSRit is the CSR performance of firm i in year t; short-termism is the managerial short-termism indicator of firm i in year t. Xit is the set of control variables, including firm size, debt, growth, gender diversity and independent directors. To isolate the impact of managerial short-termism from potential unobserved time-invariant firm characteristics, we add firm fixed effects. In addition, we include year-fixed effects ( ) in all the models to control for unobserved firm-invariance factors. Our robust standard errors are clustered at the firm level.
Next, to test the moderating effect of internal corporate governance, product market competition, analyst coverage, and ownership structure, we develop Formula (4) [142]:
(4)
where indicates whether firm i in year t is in the fractile with the lowest, the medium, or the highest corporate governance level (CGI), product market competition (HHI), and the level of analyst coverage. In addition, the ownership structure is classified into two types: state-owned and non-state-owned. The detailed estimation of each moderation variable is included in Section 3.2.3. The remaining variables are identical to those of Equation (3).
4. Results
4.1 Baseline results
We use the variance inflation factor (VIF) statistic [143] to test whether the regressions have multicollinearity issues [144]. The results show that the VIF for each independent variable is far less than 5.9 Hence, the regressions do not have multicollinearity issues. Table 2 reports the results from estimating Formula 3, where we introduce control variables in a stepwise way. Column (1) shows that an increase in managerial short-termism leads to a drop in firm CSR performance, which is consistent with previous findings [121]. Our estimates in Column (2) add financial indicators into the specification, and we obtain the robustness results. Column (3) includes information on all time-varying firm characteristics that could affect CSR performance, including both financial and management factors.
Our findings are consistent with H1b that managerial short-termism reduces CSR performance. This is because CSR is an investment that involves uncertainties in terms of its return and takes time to come to fruition [145]. The decision to invest in CSR is likely to be affected by CEOs' career concerns. As discussed earlier, the literature suggests that due to their positive future career prospects, CEOs with higher managerial short-termism are under more pressure to pursue projects that generate payoffs sooner and with greater certainty.

9 VIF for variables of short-termism, growth, size, debt, HHI, SOE, CGI, CEO, and gender are 1.02,1,28, 1.08,1.27,1.02,1.12,1.01, and 1.02, respectively.

In terms of control variables, we find that companies with higher growth, larger scale, or lower debt perform better on CSR. The effect of internal corporate governance is also statistically significant.
[Insert Table 2 here]
4.2 Endogeneity issue
We employ the two-stage residual inclusion (2SRI) model avoid endogeneity issues [146,147].
First, we use Equation (3) to estimate the influencing factors of managerial short-termism10 and then take the residual value as incremental managerial short-termism and include it in Equation (5) for regression.
(5)
To ensure that the managerial short-termism index captures managerial characteristics, on the basis of Equation (3), we further control the short-termism proxy variables, including the ratio of short investments estimated by the ratio of short investments to total assets (Short_invest), shareholder turnover rates calculated by the annual average stock turnover ratio (Turnover), and the number of earnings announcements made by management (MF) estimated by Ln (the number of company performance announcements in the current year +1).
Table 3 shows the 2SRI results. Under the endogenous hypothesis, according to the empirical judgment rule of "weak instrumental variables", if the F statistic of the first stage is greater than 10, there is no need to worry about the problem of weak instrumental variables [148]. The F statistic of this paper is greater than 10 and is obvious at the 1% statistical significance level. The Sargan statistic further indicates that the instrumental variables are exogenous (p > 10%), so the instrumental variables satisfy the correlation and exogeneity assumptions. In Column (1) and (3), we find that the coefficient

on the first-stage residuals is significantly negative at 1% significance level, indicating that the data support an endogenous effect in our model specification [146]. After alleviating endogeneity issue, we obtain the consistent results that the effect of short-termism on CSR performance is significantly negative. Therefore, our baseline results are robust.

[Insert Table 3 here]

4.3 Random forest regression

The random forest regression algorithm involves the use of ensemble learning to improve forecast accuracy [149]. It is claimed that it is not necessary to consider problems of multicollinearity as many variables can be added [150]. Moreover, we should not begin by assuming that the relationship between managerial short-termism and CSR performance is linear. Figure 1 shows that the association between managerial short-termism and CSR performance is negative.

[Insert Figure 1 here]

5. Possible channels

By the above baseline tests, we confirm that managerial short-termism has an adverse impact on CSR performance. In this section, we further explore the mechanisms by which managers' short-

10 The regression specification (4) is nonlinear because short-termism is nonnegative.

termism negatively affects their CSR behavior in China. Considering that managers are generally under pressure to perform well and immediately signal their ability to shareholders, we examine how managerial short-termism affects CSR performance via inefficient investments [151] and value-destroying acquisitions [152].

In Table 4, we change the dependent variable and employ the same empirical model as in Equation (3) to examine the possible channels. Specifically, in Column (1), the dependent variable is capital expenditure divided by annual average total assets. The positive coefficient on managerial short-termism implies that higher managerial short-termism has higher average capital expenditure.

We use acquisitions as an additional indicator to estimate short-term investments [142]. In Columns (2) and (3) of Table 4, based on the literature, we further investigate the channel by examining the acquisition likelihood and the number of acquisitions in a given year. In Column 2, acquisition likelihood is a dummy variable that equals one if a firm merges in a given year and zero otherwise. In Column (3), the dependent variable acquisition number is calculated by Ln (acquisition number +1). Columns (2) and (3) show significant positive coefficients of managerial short-termism. According to the above results and agency theory, managers are prone to make inefficient investments and value-destroying acquisitions due to careen concerns and then reduce long-term CSR activities.

[Insert Table 4 here]

6. Heterogeneity Checks

We further illustrate the impact of supervisory factors such as the level of internal corporate governance, product market competition (external governance), analyst coverage and ownership structure.

The results in Models (1) to (4) show that the VIF for each independent variable is less than 5 (see Appendix A Table 2). Therefore, the four heterogeneity checks are without multicollinearity issues.

The results of Equation (4) are presented in Table 5. In Column (1), we find that the coefficient of short-termism is significantly negative at the 1% significance level, and only the coefficient of the interactive term of “ ” is significantly negative. This indicates that the adverse effect of managerial short-termism is only significant for firms with poor internal corporate governance. Corporate success requires considering the interests of stakeholders beyond shareholders [98]. Following this logic, effective corporate governance helps alleviate the adverse effect of managerial short-termism on CSR performance.

In Column (2), only the coefficient of the interactive term of “ ” is significantly negative, which indicates that the adverse effect of managerial short-termism is only significant for firms in less competitive industries. Firms in competitive industries have incentives to engage more in socially responsible activities to gain a competitive edge [153]. Following this logic, higher product market competition helps alleviate the adverse effect of managerial short-termism on CSR performance.

In Column 3, only the coefficient of “ ” is significantly negative, which indicates that the adverse effect of managerial short-termism is significant only for firms lacking analyst coverage. Analyst coverage can reduce information asymmetry [154]. An increase in analyst

coverage heightens media coverage, which in turn increase firms' CSR information exposure to stakeholders. Following this logic, higher analyst coverage helps alleviate the adverse effect of managerial short-termism on CSR performance.

The results in Column 4 report that the negative impact of managerial short-termism on CSR performance is pronounced for SOEs. It because that the agency issue is more serious for SOEs, the higher information asymmetry makes managers are likely to pursue self-interest.

[Insert Table 5 here]

7. Further analysis

The most significant factor influencing the intensity of communication about CSR is the governance environment in a country [155,156]. Institutional factors have a crucial impact on a company's CSR performance [157]. Considering the wide variation in regional institutional environments across regions in China, we evaluate the impact of regional institutional environments on the association between managerial short-termism and CSR performance:

(6)

where institution refers to the institutional environment. We measure the regional institutional environment using the method as , where market represents the regional marketization index and is an indicator of regional market segmentation. Table 6 presents the results for Eq. (6). It shows that managerial short-termism reduces CSR performance. In Column 1, we find statistically significant and negative estimates for managerial short-termism. The coefficient of is positive and significant at the 1% level. The results indicate that a good institutional environment enables a decrease in the adverse effect of managerial short-termism on CSR performance. In Column 2 of Table 6, we control the firm characteristic variables that may influence CSR performance in our specification, and we find that the coefficient on the interactive term of is still significantly positive at the 0.01 level. The coefficient of remains robust when the year and company fixed effects are considered. A positive institutional environment weakens the negative impact of managerial short-termism on CSR performance.

[Insert Table 6 here]

8. Conclusions, policy implications and limitations

8.1 Conclusions

In conjunction with time-oriented theory, agency theory and the high-level echelon theory of social psychology, we test the effect of managerial short-termism on CSR performance. First, we construct the indicator of managerial short-termism by using textual analysis and machine learning methods. Second, from the perspective of managers' internal characteristics, we theoretically and empirically test the impact of managerial short-termism on enterprises' CSR performance. The results show that (1) when MD&A discloses more 'short-termism' words, it indicates that managers have the characteristics of short-term time orientation. (2) Managerial short-termism reduces CSR performance.

(3) The negative impact of managerial short-termism on CSR performance is significant only for firms with lower internal corporate governance, for firms in less competitive industries, for firms with less analyst coverage and for SOEs. (4) The negative impact of managerial short-termism on CSR performance is weakened by a high-quality institutional environment.

8.2 Policy implications

The core conclusions of this paper have some policy implications.

First, this paper figures out that managerial short-termism reduces CSR performance. When selecting and training senior managers, enterprises should pay attention not only to their demographic characteristics but also to the characteristics of managers' time orientation.

Second, the results show that the negative impact of managerial short-termism on CSR performance significant only for firms with lower internal corporate governance, for firms in less competitive industries, for firms with less analyst attention, and for SOEs. That is, it confirms the inhibitory effect of internal and external supervision on managers' short-termism. Enterprises should further improve their internal corporate governance, give full play to the internal supervision mechanism, and comprehensively improve their corporate governance ability.

Third, the governments should improve the quality of institutional environments like, strengthening of formal market systems.

8.3 Limitations

There are two limitations in this paper. First, we only focus on listed companies, which leads to a limited observations. We hope future research can also focus on the CSR of unlisted industrial enterprises. Second, we did not classify the different types of CSR activities. We suggest that future research extend this work by breaking down the CSR score into separate social, environmental, and governance indicators. Third, we did not take into consideration the foreign ownership structure in this paper. This study can be extended to compare SOEs, domestic private enterprises and foreign ownership enterprises. Moreover, though we distinguish the motivations for CSR in theoretical analysis, the we did not test is empirically in this paper. Future studies could examine the impact of motivities for CSR empirically.

Appendix A

The key words in Chinese used to estimate managerial short-termism in MD&A files are presented in the following Table.

Appendix Table 1 Words in Chinese used to estimate managerial short-termism index

(1) 种子词<br>词集	天内、数月、年内、尽快、立刻、马上、契机、之际、压力、考验
(2) 扩充词<br>词集	日内、数天、随即、即刻、在即、最晚、最迟、关头、恰逢、来临之际、前夕、适逢、遇上、正逢、之时、难度、困境、严峻考验、双重压力、通胀压力等

Appendix Table 2 Variables definition and measurement

Variable	Sign	Measurement
Corporate social responsibility	CSR	the logarithm of CSR performance

Variable	Sign	Measurement
Managerial short-termism	Short-termism	the proportion of the total word frequency of 'short-termism' vocabulary in the total word frequency of MD&A, and multiplies it by 100
Firm growth	Growth	the net profit growth
Firm size	Size	the logarithm of firm total assets
Firm Debt	Debt	The ratio of total liabilities by shareholder equity
Product market competition	HHI	the sum of the sales-based square market shares of all firms in an industry
Ownership	SOE	a dummy variable, if it is SOEs coded as "1"
Corporate governance	CGI	derived from (Yu et al., 2017)'s corporate governance index CGI
Independent director	CEO	a dummy variable, when the CEO and the chairman are the same, the value is 1; otherwise, 0
Gender diversity	Gender	a dummy variable for female chairman coded as "1", otherwise, coded as "0"

Appendix B

Table 1 Descriptive statistics of main variables

	Mean	Median	Max	Min	Std. Dev	Obs.
CSR	3.62	3.59	4.34	2.92	0.30	4913
Short-termism	0.11	0.09	0.76	0.00	0.09	4913
Growth	0.24	0.20	0.95	0.00	0.19	4913
Size	23.12	22.96	29.33	18.27	1.49	4913
Debt	0.50	0.49	1.66	-0.51	0.20	4913
HHI	0.10	0.07	0.65	0.02	0.10	4913
SOE	0.64	1.00	1.00	0.00	0.48	4913
CGI	48.81	48.72	59.46	37.04	5.02	4913
CEO	0.14	0.00	1.00	0.00	0.35	4913
Gender	0.08	0.00	1.00	0.00	0.27	4913

Descriptions of the major variables in the paper are shown in this table. The measurements of variables

are in Table 1.

Table 2 Baseline results

(1) (2) (3) (4)

Short-termism -0.103** -0.102** -0.091** -0.087**

(0.04) (0.04) (0.04) (0.04)

Growth 0.018*** 0.020***

(0.00) (0.00)

Size 0.030*** 0.031***

(0.00) (0.00)

Debt -0.101*** -0.108***

(0.02) (0.02)

HHI -0.128* -0.101

(0.07) (0.07)

SOE 0.041*** 0.044***

(0.01) (0.01)

CGI 0.008*** 0.009***

(0.00) (0.00)

CEO -0.028*** -0.011

(0.01) (0.01)

Gender -0.004 -0.001

(0.01) (0.01)

Firm cluster Yes Yes Yes Yes

Firm fixed Yes Yes Yes Yes

Year fixed Yes Yes Yes Yes

_Cons 4.273*** 3.587*** 3.940*** 3.195***

(0.02) (0.03) (0.04) (0.04)

N4913 4913 4913 4913

Adjust

0.30 0.32 0.32 0.34

Note: The table provides information on the impact of managerial short-termism on the firms’ CSR performance. The measurements of variables are in appendix Table

2. Within the top and bottom 1% of their distributions, we winorize all continuous variables. 'Yes' indicates that we control the fixed item in this model, otherwise 'No'.

*, **, *** indicate statistical significance at the 10%, 5%, and 1% levels, respectively. The robust standard errors are in parentheses. The period covered is from 2008 to 2017.

Table 3 robustness checks: endogeneity issue

(1) (2) (3) (4)

Residual(t) -0.061***

(0.02)

Residual(t-1) -0.064***

(0.02)

Short_invest 0.046* -0.008 0.051* -0.007

(0.03) (-0.65) (0.02) (-0.67)

Control variables Yes Yes Yes Yes

Firm cluster Yes Yes Yes Yes

Company Fixed Yes Yes Yes Yes

Year Fixed Yes Yes Yes Yes

_Cons 0.043* 3.288*** 3.000*** 3.193***

(0.02) (0.04) (0.03) (0.04)

N4912 4912 4827 4827

Adjust 0.13 0.34 0.11 0.34

Wald-test 76.80*** 77.21***

0.00 0.00

F-test 59.28*** 65.37***
0.00 0.00
Sargan statistic 1.51 1.62
0.22 0.23
Note: The table provides information on the2SLS regressions results. The measurements of variables are in appendix Table 2. Within the top and bottom 1% of their distributions, we winorize all continuous variables. 'Yes' indicates that we control the fixed item in this model, otherwise 'No'. Short_invest is calculated by the ratio of short investments to total assets. Turnover is calculated by annual average stock turnover ratio. MF is estimated by Ln (the number of company performance announcements in the current year +1). *, **, *** indicate statistical significance at the 10%, 5%, and 1% levels, respectively. The robust standard errors are in parentheses. The period covered is from 2008 to 2017.
Figure 1 the association between managerial short-termism and CSR performance
Table 4 Channels test
Capital
expenditure
(1)
Acquisition
likelihood
(2)
Acquisition
number
(3)
Short-termism 0.024*** 2.275*** 0.205***
(0.00) (0.36) (0.05)
Control variables Yes Yes Yes
Company
dummies
Yes Yes Yes
Year dummies Yes Yes Yes
Observations 4913 4913 4913
R-squared 0.001 0.026
Pseudo R-
squared
0.010
Note: The table provides information on the channels though which managerial short-termism influences the firms' CSR performance. The measurements of variables are in appendix Table 2.Within the top and bottom 1% of their distributions, we winorize all continuous variables. 'Yes' indicates that we control the fixed item in this model, otherwise 'No'. *, **, *** indicate statistical significance at the 10%, 5%, and 1% levels, respectively. The robust standard errors are in parentheses. The period covered is from 2008 to 2017. In addition, the result in column is from the logit regression.

Table 5. Heterogeneity checks: firm-level heterogeneity
CGI
(1)
HHI
(2)
Analyst coverage
(3)
Ownership structure
(4)
-0.301***
(0.02)
-0.210
(0.24)

-0.109
(0.12)
0.120
(0.12)
-0.123***
(0.02)
-0.330***
(0.10)
0.036
(0.00)
0.048
(0.11)
-0.031***
(0.02)
-0.032
(0.12)
-0.039
(0.12)
Control variables Yes Yes Yes Yes
Company Fixed Yes Yes Yes Yes
Year Fixed Yes Yes Yes Yes
_Cons 3.341*** 3.420*** 3.332*** 3.344***
(0.09) (0.05) (0.05) (0.04)
N4913 4913 4913 4913
Adjust 0.06 0.08 0.14 0.08

Note: The table provides information on heterogeneity effects. The measurements of variables are in appendix Table 2.Within the top and bottom 1% of their distributions, we winorize all continuous variables. 'Yes' indicates that we control the fixed item in this model, otherwise 'No'. *, **, *** indicate statistical significance at the 10%, 5%, and 1% levels, respectively. The robust standard errors are in parentheses. The period covered is from 2008 to 2017.

Table 6. The impact of institutional environment

(1) (2) (3)
Short-termism -0.044*** -0.284** -0.312***
(0.01) (0.13) (0.08)
0.059** 0.067*** 0.069***
(0.02) (0.01) (0.01)
Institution 0.049*** 0.055*** 0.022***
Control variables No Yes Yes
Company Fixed No No Yes
Year Fixed No No Yes
_Cons 3.662*** 3.174*** 3.349***
(0.02) (0.05) (0.06)
N4156 4156 4156
Adjust
0.01 0.08 0.38

Note: The table provides information on the role of institutional environment. The measurements of variables are in appendix Table 2. Within the top and bottom 1% of their distributions, we winorize all continuous variables. 'Yes' indicates that we control the fixed item in this model, otherwise 'No'. *, **, *** indicate statistical significance at the 10%, 5%, and 1% levels, respectively. The robust standard errors are in parentheses. The period covered is from 2008 to 2017.
